# FPGA-Based On-Board Geometric Calibration for Linear CCD Array Sensors

**DOI:** 10.3390/s18061794

**Published:** 2018-06-02

**Authors:** Guoqing Zhou, Linjun Jiang, Jingjin Huang, Rongting Zhang, Dequan Liu, Xiang Zhou, Oktay Baysal

**Affiliations:** 1 Guangxi Key Laboratory for Spatial Information and Geomatics, Guilin University of Technology, Guilin 541004, China; zqx0711@tju.edu.cn; 2 School of Precision Instrument & Opto-Electronics Engineering, Tianjin University, Tianjin 300072, China; jingjin_huang@tju.edu.cn (J.H.); zrt65@tju.edu.cn (R.Z.); 3 The Center for Remote Sensing, Tianjin University, Tianjin 300072 China; 4 Guangxi Institute of Geoinformation, Surveying and Mapping, Liuzhou 545006, China; linjunastro@163.com; 5 School of Microelectronics, Tianjin University, Tianjin 300072, China; ldqzhh@tju.edu.cn; 6 Mechanical & Aerospace Engineering, Old Dominion University, Norfolk, VA 23529, USA; obaysal@odu.edu

**Keywords:** FPGA, on-board, geometric calibration, parallel computing, spaceborne sensor

## Abstract

With increasing demands in real-time or near real-time remotely sensed imagery applications in such as military deployments, quick response to terrorist attacks and disaster rescue, the on-board geometric calibration problem has attracted the attention of many scientists in recent years. This paper presents an on-board geometric calibration method for linear CCD sensor arrays using FPGA chips. The proposed method mainly consists of four modules—Input Data, Coefficient Calculation, Adjustment Computation and Comparison—in which the parallel computations for building the observation equations and least squares adjustment, are implemented using FPGA chips, for which a decomposed matrix inversion method is presented. A Xilinx Virtex-7 FPGA VC707 chip is selected and the MOMS-2P data used for inflight geometric calibration from DLR (Köln, Germany), are employed for validation and analysis. The experimental results demonstrated that: (1) When the widths of floating-point data from 44-bit to 64-bit are adopted, the FPGA resources, including the utilizations of FF, LUT, memory LUT, I/O and DSP48, are consumed at a fast increasing rate; thus, a 50-bit data width is recommended for FPGA-based geometric calibration. (2) Increasing number of ground control points (GCPs) does not significantly consume the FPGA resources, six GCPs is therefore recommended for geometric calibration. (3) The FPGA-based geometric calibration can reach approximately 24 times faster speed than the PC-based one does. (4) The accuracy from the proposed FPGA-based method is almost similar to the one from the inflight calibration if the calibration model and GCPs number are the same.

## 1. Introduction

Geometric calibration is one of the most important steps for quality control in high-resolution optical satellite imagery [1,2,3] and is a prerequisite for high-accuracy of direct georeferencing of remotely sensed images [4,5]. Previous researchers have made efforts in inflight geometric calibration on the basis of PC computers in the past decades. With increasing demands for real-time or near real-time remotely sensed imagery in applications such as military deployments, quick response to terrorist attacks and disaster rescue (e.g., flooding monitoring), the on-board implementation of geometric calibration is has been attracting many scientists’ interest worldwide in recent years.

The parameters of geometric calibration consist of the exterior orientation elements (EOEs) and interior orientation elements (IOEs). Usually, the IOEs are calibrated in the laboratory, while the EOEs often change due to micro-gravidity, solar pressure, etc. when the satellite runs in space for a certain while. Hence it is necessary to develop the calibration method and algorithm to carry out an on-board geometric calibration.

**Figure 1:**
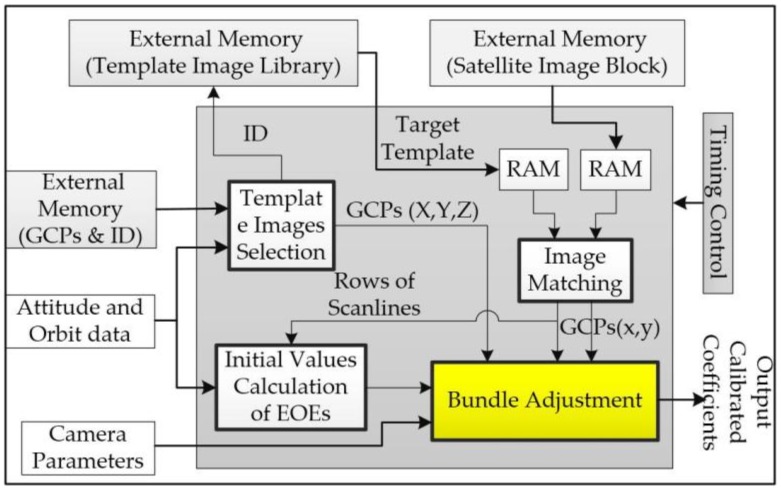
The FPGA architecture of geometric calibration.

The concept of “on-board geometric correction” was first presented by Zhou et al. [6], but the authors did not present any details of its on-board implementation. This paper presents a Field Programmable Gate Array (FPGA)-based implementation of geometric calibration. The FPGA can offer a highly flexible design, a scalable circuit, and a high efficiency in data processing, because of its pipeline structure and fine-grained parallelism. Moreover, the DSP units embedded in the FPGA are suitable for floating point arithmetic. The proposed FPGA-based architecture of geometric calibration is depicted in Figure 1, which consists of five modules: Template Image Selection, Image Matching (Huang and Zhou [7]), Initial value EOEs, Bundle Adjustment, and Timing Control.

All of the functional modules in Figure 1 are managed by the Timing Control module. The Template Images Selection module is used for selection of target template images from a template image library that is created from many template images, each of which has a unique ID number. The principle of this module is carried out through matching between the geodetic coordinates centralized at an imaged area and the coordinates of a georeferenced template image. The Image Matching module accurately determines the coordinates of spaceborne image and geodetic coordinates of template images by matching their sub-image windows. The Initial external orientation elements (EOEs) module computes the initial values of EOEs of the spaceborne sensor. The Bundle Adjustment module accurately computes six external orientation elements (EOEs). Due to the resource limitation of a FPGA chip, it usually applies external memory to store multiple template images and ground control points (GCPs) data. Random Access Memory (RAM) is used to store the data stream of a template image and/or an imaged scene temporarily. The line buffers of image data are generated using multiple RAMs for further image matching.

This paper presents the details of FPGA-based implementation of on-board geometric calibration. The input parameters (i.e., GCPs and initial EOEs) are assumed to be directly provided by other modules (for the details, please reference to the Huang and Zhou [7]). The paper is organized as follows: Section 2 overviews previously relevant efforts; Section 3 gives the detailed FPGA-based implementation of on-board geometric calibration. Section 4 describes the validation and the experimental results. The conclusions are drawn up in Section 5.

## 2. Relevant Efforts

Traditional geometric calibration methods have been investigated for several decades and a number of papers have been published in the computer vision [8,9,10], image processing [11,12], robotic vision [13,14] and photogrammetry communities. However, geometric calibration on-board spaceborne implementation has not yet been reported worldwide so far, although in-lab and/or inflight (also called *on-orbit*) geometric calibrations for various satellites have overwhelmingly been reported in the past two decades [15,16,17], such as SPOT1-5 [18], IKONOS [19], ALOS [20], Orbview-3 [21], IRS-1C [22], GeoEye-1 [23], MOMS-2P [24,25], CBERS-02B [26], TH-1 [27] and ZY-3 [28,29].

Jacobsen [30,31] implemented geometric calibration of the IRS-1C satellite using self-calibration bundle block adjustment with additional parameters. Crespi et al. [23] used the block adjustment method to investigate the inflight calibration of the GeoEye-1 satellite, with which the accuracy reached 3.0 m without GCPs. Lei [28] presented a self-calibration bundle block adjustment on the basis of the known interior orientation parameters with line array CCD. Li [29] and Li et al. [32] proposed a geometric calibration method with step-by-step on the basis of imaging model of the ZY-3 satellite. The experimental results indicated that the calibration accuracy of ZY-3 can meet the requirement of the accuracy of 1:50,000 mapping. Yang et al. [33] also presented an on-orbit geometrical calibration model for ZY-1 02C panchromatic camera. The experimental results demonstrated that the accuracy with or without GCPs is better than 0.3 pixels. Zhang et al. [34] proposed an on-orbit geometric calibration for and a validation of ZY-3 linear array sensors. Wang et al. [3] constructed an on-orbit rigorous geometric calibration model through selecting optimal parameters. The experimental results discovered that the geometric accuracy without GCPs is significantly improved after on-orbit geometric calibration. Cao et al. [35] proposed a named “*look-angle calibration method*” for on-orbit geometric calibration of ZY-3 satellite imaging sensors. The experimental analysis discovered that the accuracy under the nadir-looking images is higher than ±2.7 m when five GCPs are utilized and the laboratory calibration parameters are provided as initials. Cao et al. [36] proposed a simple and feasible orientation method for calibration of the CCD-detector’s look angles in the three-line array cameras of ZY-3. The experimental results discovered that the accuracies in planimetry and height are 3.4 m and 1.6 m with four GCPs, respectively. Wang et al. [37] proposed an on-orbit geometric calibration method for TH-1 three-line-array camera based on a rigorous geometric model. The experiment results demonstrated that the accuracies in both planimetry and height are approximately 6.93 m and 3.96 m, respectively.

Although research in on-board geometric calibration is relatively rare so far, the FPGA-based on-board data processing for spaceborne images has been reported. For instance, the German small satellite BIRD applied an on-board data processing system consisting of DSP, FPGA and network co-processor in on-board implementation of radiation correction, rectification of partial systematic geometric and image classification using neural network algorithm [38]. Surrey Satellite Technology Limited in the UK applied FPGA as an on-board data processing chip in a small satellite [39]. The French used FPGA chip to realize on-board data processing for Pleiades imagery [40]. Stuttgart University in Germany designed an on-board computing system using FPGA for the small satellite Flying Laptop to realize real-time attitude control, housekeeping, data compression and image processing [41]. The U.S. Jet Propulsion Laboratory employed a Xilinx FPGA to realize on-board hyperspectral image classification based on Support Vector Machine (SVM) [42]. González et al. [43] conducted the investigation on an FPGA-based implementation of N-FINDR algorithm for hyperspectral image analysis. Williams et al. [44] also investigated an FPGA-based implementation of real-time cloud detection of spaceborne image. Hihara et al. [45] analyzed an on-board image processing system for hyperspectral imagery. All of the efforts mentioned above have shown a promise for FPGA-based implementation of geometric calibration, which is presented in this paper.

## 3. On-Board Geometric Calibration Using FPGA Chip

### 3.1. Brief Overview of Geometric Calibration Model

The different linear imaging systems may have their own imaging modes, resulting in the slight difference of the geometric calibration algorithm. This paper takes MOMS-2P as an example to describe the calibration model (for details readers should refer to [46,47]).

The geometric calibration algorithm of the MOMS imaging system was performed at the laboratories of the German Aerospace company DASA (Stuttgart, Germany), where the MOMS was developed and manufactured. A rigorous model of describing geometry calibration is composed of five parameters (also see Table 1):

Principal point coordinates of each sensor (*x*_0_, *y*_0_),
Rotation parameter *κ* of CCD array in the image plane,

Deviation of the focal length *df* and distortion parameter *k* of the sensor curvature. The sensor curvature is modeled by a second order polynomial equation. The parameter *k* here indicates the along track deviation at the edges of the CCD-array at 3000 pixel distance from the array center, caused by the sensor curvature.

#### 3.1.1. Interior Orientation

**Figure 2:**
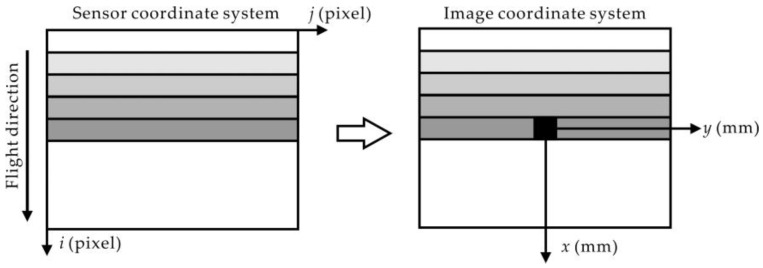
Sensor/screen and image coordinate system.

Interior orientation is to transform sensor coordinates (*i* and *j* in Figure 2) into image coordinates (*x* and *y* in Figure 2) and correct the lens distortion (symmetric and tangential) and CCD array curvature distortion. Thus the following tasks should be performed.

- Define the sensor (called “*screen*”) coordinate system and image coordinate systems,
- Apply the principal point offset that should be estimated from an in-lab or in-flight calibration, and
- Correct various distortions.

#### 3.1.2. Transformation from Image to Reference Coordinate System

**Figure 3:**
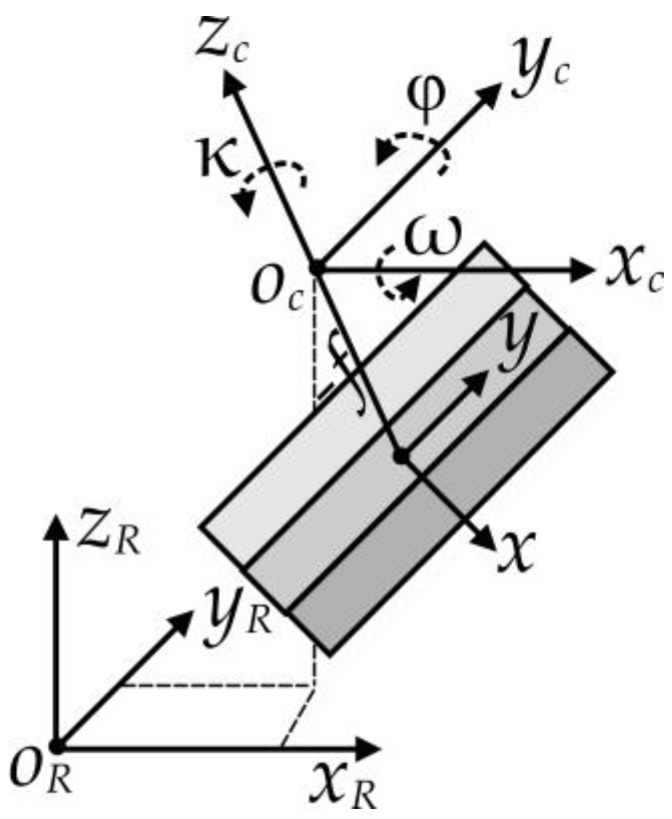
From image coordinate to image reference coordinate system.

Each of the fore-, nadir- and aft-looking array has its own image coordinate system (*x_c_*, *y_c_* and *z_c_*, right-handed). An image reference coordinate system (*x_R_*, *y_R_* and *z_R_*, right-handed) is defined to unify image coordinates from all three arrays (Figure 3). The transformation from an image coordinate system to the reference coordinate system involves a translation (*d_x_*, *d_y_* and *d_z_*) and three rotations (*ω*, *φ* and *κ*). We define a counterclockwise rotation angle as positive. The transformation equation is:

(1) $$\left(\begin{array}{c} x_{R}\\ y_{R}\\ z_{R} \end{array}\right)=R_{C}^{R}\left(\begin{array}{c} x_{c}\\ y_{c}\\ z_{c} \end{array}\right)+\left(\begin{array}{c} d_{x}\\ d_{y}\\ d_{z} \end{array}\right)=R_{C}^{R}\left(\begin{array}{c} x\\ y\\ -f \end{array}\right)+\left(\begin{array}{c} d_{x}\\ d_{y}\\ d_{z} \end{array}\right)$$

where:

(2) $$R_{C}^{R}=\left(\begin{array}{ccc} cos\varphi cos\kappa & cos\omega sin\kappa +sin\omega sin\varphi cos\kappa & sin\omega sin\kappa -cos\omega sin\varphi cos\kappa \\ -cos\varphi sin\kappa & cos\omega cos\kappa -sin\omega sin\varphi sin\kappa & sin\omega cos\kappa +cos\omega sin\varphi sin\kappa \\ sin\varphi & -sin\omega cos\varphi & cos\omega cos\varphi \end{array}\right)$$

#### 3.1.3. Geometric Calibration Model

For any image point within a CCD array, its image reference coordinates are (*x_R_*, *y_R_* and *z_R_*). The coordinates of the exposure center of the array in the ground coordinate system at the imaging epoch *t* are (*X_C_*(*t*), *Y_C_*(*t*), *Z_C_*(*t*)). The corresponding ground point coordinates are (*X_G_*, *Y_G_*, *Z_G_*). The collinearity condition states that all of the three points must lie on the same straight line:

(3) $$\{\begin{array}{c} x_{R}=z_{R}\frac{r_{11}(X_{G}-X_{C}(t))+r_{12}(Y_{G}-Y_{C}(t))+r_{13}(Z_{G}-Z_{C}(t))}{r_{31}(X_{G}-X_{C}(t))+r_{32}(Y_{G}-Y_{C}(t))+r_{33}(Z_{G}-Z_{C}(t))}\\ y_{R}=z_{R}\frac{r_{21}(X_{G}-X_{C}(t))+r_{22}(Y_{G}-Y_{C}(t))+r_{23}(Z_{G}-Z_{C}(t))}{r_{31}(X_{G}-X_{C}(t))+r_{32}(Y_{G}-Y_{C}(t))+r_{33}(Z_{G}-Z_{C}(t))} \end{array}$$

where *r_ij_* (*i*, *j* = 1, 2, 3) are the elements of rotation matrix $R_{G}^{R}=R_{C}^{R}(\varphi (t),\omega (t),\kappa (t))$; *φ*(*t*), *ω*(*t*) and *κ*(*t*) are defined for each CCD array at the epoch *t*.

A separate image coordinate system is defined for each CCD array which is related to an image reference coordinate system by a 3D transformation. They are a focal length *f*, principal point offset (*x*_0_, *y*_0_), and a curvature parameter *Kc*. The sensor coordinates (*i*, *j*) are transformed to image coordinates in the following steps (the details can be referenced to [46,47]):

- *Step 1*: Transformation from sensor/screen coordinates to image coordinates by $x^{\prime }=0$; $y^{\prime }=(j-column/2)\times 10e-3(\mathrm{mm})$;
- *Step 2*: CCD curvature correction by $cx=Kc\times y^{\prime }\times y^{\prime }$;
- *Step 3*: Lens distortion correction is unavailable;
- *Step 4*: Final image coordinates computed by $x=cx-x_{0}$, $y=y^{\prime }-y_{0}$.

To reduce the calculation budget and save the FPGA resources, a first-order polynomial equation is adopted to compute the six EOEs, i.e.:

(4) $$\left\{ \begin{array}{c} X_{C}(t)=X_{C}^{0}+a_{0}\;\;\varphi_{C}(t)=\varphi_{C}^{0}+d_{0}+d_{1}t\\ Y_{C}(t)=Y_{C}^{0}+b_{0}\;\;\omega_{C}(t)=\omega_{C}^{0}+e_{0}+e_{1}t\\ Z_{C}(t)=Z_{C}^{0}+c_{0}\;\;\kappa_{C}(t)=\kappa_{C}^{0}+f_{0}+f_{1}t \end{array}\right.$$

where $\left(X_{C}^{0}(t),Y_{C}^{0}(t),Z_{C}^{0}(t),\varphi_{C}^{0}(t),\omega_{C}^{0}(t),\kappa_{C}^{0}(t)\right)$ are initial values of EOEs at epoch *t*. The initial values of EOEs are provided by DLR, Germany. In one epoch *t*, Equation (3) is linearized by Taylor Series. Substitute Equation (4) into Equation (3), and then linearize it by Taylor Series, it yields:

(5) $$\left\{ \begin{array}{c} v_{x}=a_{11}\Delta a_{0}+a_{12}\Delta b_{0}+a_{13}\Delta c_{0}+a_{14}\Delta d_{0}+a_{15}\Delta e_{0}+a_{16}\Delta f_{0}+a_{17}\Delta d_{1}+a_{18}\Delta e_{1}+a_{19}\Delta f_{1}-l_{x}\\ v_{y}=a_{21}\Delta a_{0}+a_{22}\Delta b_{0}+a_{23}\Delta c_{0}+a_{24}\Delta d_{0}+a_{25}\Delta e_{0}+a_{26}\Delta f_{0}+a_{27}\Delta d_{1}+a_{28}\Delta e_{1}+a_{29}\Delta f_{1}-l_{y} \end{array}\right.$$

The vector form of Equation (5) is described as:

(6) $$V_{t}=A_{t}X_{t}-l_{t}$$

where $V_{t}={\left[\begin{array}{cc} v_{x} & v_{y} \end{array}\right]}^{T}$, $X_{t}={\left[\begin{array}{ccccccccc} \Delta a_{0} & \Delta b_{0} & \Delta c_{0} & \Delta d_{0} & \Delta e_{0} & \Delta f_{0} & \Delta d_{1} & \Delta e_{1} & \Delta f_{1} \end{array}\right]}^{T}$, which are the unknowns; $l_{t}={\left[l_{x}l_{y}\right]}^{T}$; *A_t_* is coefficient vector, which is expressed by:

(7) $$A_{t}=\left[\begin{array}{ccccccccc} a_{11} & a_{12} & a_{13} & a_{14} & a_{15} & a_{16} & a_{17} & a_{18} & a_{19}\\ a_{21} & a_{22} & a_{23} & a_{24} & a_{25} & a_{26} & a_{27} & a_{28} & a_{29} \end{array}\right]$$

The detailed derivation of *A_t_* can be found in [46,47]. When the number of GCPs are greater than 5, the observation equation is constructed as follows:

(8) V=AX−L

where *V =* [*V*_1_ *V*_2_ L *V_n_*]*^T^*, *X =* [*X*_1_ *X*_2_ L *X_n_*]*^T^*, *A =* [*A*_1_ *A*_2_ L *A_n_*]*^T^*, *L =* [*l*_1_ *l*_2_ L *l_n_*]*^T^*, *n* represents the number of GCPs. The Equation (8) is solved by least square algorithm, and the solutions are:

(9) $$X={\left(A^{T}A\right)}^{-1}\left(A^{T}L\right)$$

**Figure 4:**
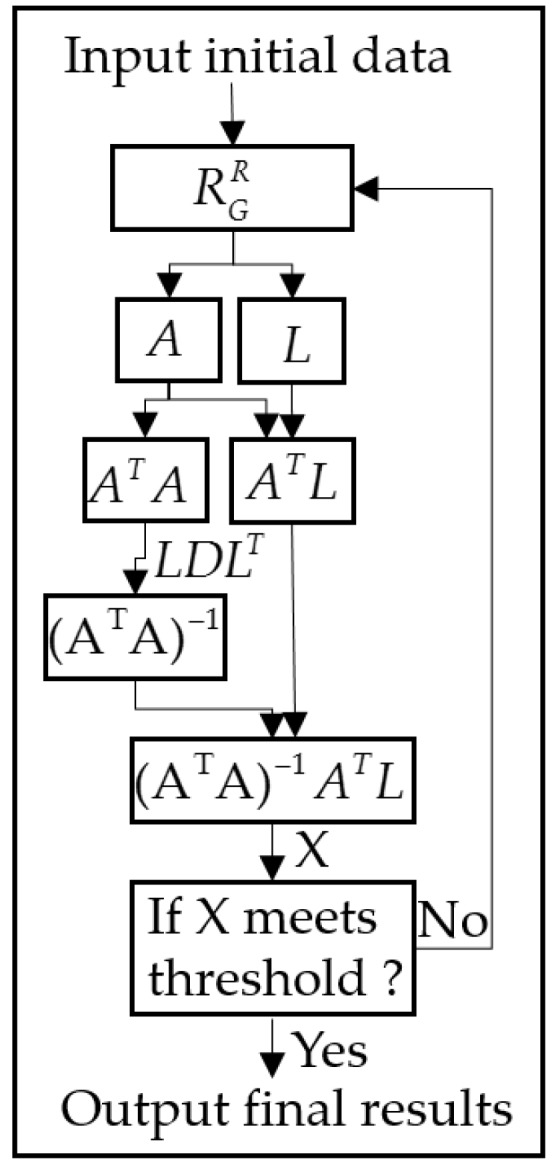
Flowchart of the whole algorithm.

The solution for Equation (9) is obtained by an iterative process. *A* flowchart of the entire algorithm is presented in Figure 4. 

As seen from Figure 4, the initial data are first used to compute the rotate matrix ($R_{G}^{R}$), which are used for computation of the coefficient matrix, *A* and constant matrix, *L*. The inversion of (*A^T^A*) is computed by an *LDL^T^* algorithm. The solution, *X*, is obtained by the iteration process and is compared to a given threshold. If the increments of *X* are less than the given threshold, then computation ends, and the *X* is considered as the final solution. Otherwise, the above computation is repeated, until the increments are less than the given threshold.

### 3.2. FPGA-Based Computation of On-Board Geometric Calibration

#### 3.2.1. Design for On-Board Geometric Calibration

**Figure 5:**
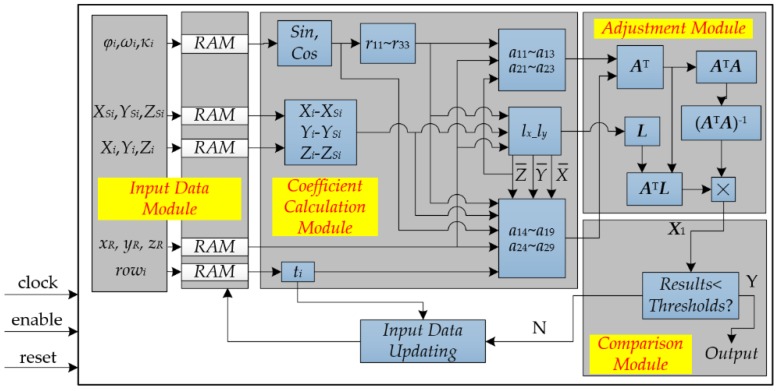
The proposed implementation of on-board geometric calibration.

An FPGA-based implementation for on-board geometric calibration is proposed in Figure 5, which consists of four modules: Input Data, Coefficient Calculation, Adjustment Computation, and Comparison. The details of the four modules are described as follows:

(1) The initial data and the updating data are stored in RAM of the Input Data module. When receiving an enable signal, the data are sent to the Coefficient Calculation module at the same clock cycle.
(2) The elements of matrixes *A* and *L* (in Equation (8)) are calculated by the Coefficient Calculation module, and the computed results are sent to the Adjustment Computation module at the same clock cycle.
(3) The solution *X* in Equation (9) is calculated by matrixes *A* and *L* in the Adjustment Computation module.
(4) If the increments of solution *X* meet the requirement of a given threshold, the iteration computation is terminated, and the solution *a*_0_, *b*_0_, *c*_0_, *d*_0_, *e*_0_, *f*_0_, *d*_1_, *e*_1_ and *f*_1_ are outputted. Otherwise, the *X* is updated and the iteration is recomputed until the increments of the solutions meet the requirement of the given threshold.

#### 3.2.2. FPGA-Based Parallel Computation for Matrixes $R_{G}^{R}$, *l_t_* and *A_t_*

**Figure 6:**
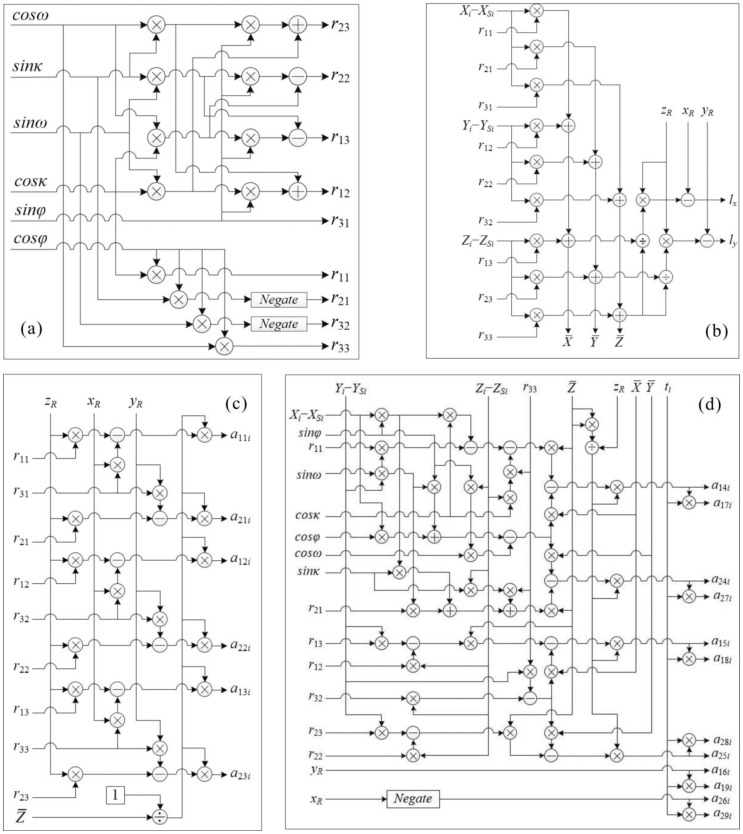
FPGA-based coefficients computation. (**a**) is for $R_{G}^{R}$ computation, *r*_11_ to *r*_33_, in which the negate unit means the negation of value; (**b**) is for *l* computation, *lx* and *ly*; (**c**) is for *a*_11_ thru *a*_13_ and *a*_21_ thru *a*_23_, computation, and (**d**) is for parallel computation of *a*_14_ thru *a*_19_ and *a*_24_ thru *a*_29_.

Because the elements (*r*_11_ through *r*_33_) in the rotation matrix $R_{G}^{R}$ involves *sine* and *cosine* functions of three rotational angles, *φ*, *ω* and *κ*, which is time/resources-consuming, a parallel computation method is presented in Figure 6a, where the implementations of *sine* and *cosine* functions are carried out by a CORDIC IP core. To ensure that all of the intermediate results are outputted at the same clock cycle, the delay units are adopted for some intermediate results. This module includes 12 multipliers, 2 adders and 2 subtractors.

For computation of *l_t_* in Equation (6), the initial data stored in the Input Data module and the rotation matrix are used to compute the *l_x_* and *l_y_*, which are presented in Figure 6b. The delay units are used to ensure that the final results are outputted at the same clock cycle.

Six elements in matrix *A_t_*, i.e., *a*_11*i*_, *a*_12*i*_, *a*_13*i*_, *a*_21*i*_, *a*_22*i*_, *a*_23*i*_, are computed in parallel, where 18 multipliers, 1 divider and 6 subtractors are employed (see Figure 6c). The rest of the elements in matrix *A_t_*, i.e., *a*_14*i*_, *a*_15*i*_, *a*_16*i*_, *a*_17*i*_, *a*_18*i*_, *a*_19*i*_, *a*_24*i*_, *a*_25*i*_, *a*_26*i*_, *a*_27*i*_, *a*_28*i*_, *a*_29*i*_, are also computed in parallel, which includes 39 multipliers, 10 subtractors, 3 adders, and 1 divider (see Figure 6d).

#### 3.2.3. FPGA-Based Parallel Computations for *A^T^A* and *A^T^L*

Due to limitation of the FPGA resource, a parallel computation method for *A^T^A* in Equation (9) is presented through modifying *A^T^A*, i.e.:

(10) $$B_{9\times 9}=A_{9\times 2n}^{T}A_{2n\times 9}=\left[\begin{array}{cccc} A_{1}A_{1} & A_{1}A_{2} & \cdots & A_{1}A_{9}\\ A_{2}A_{1} & A_{2}A_{2} & \cdots & A_{2}A_{9}\\ \vdots & \vdots & \ddots & \vdots \\ A_{9}A_{1} & A_{9}A_{2} & \cdots & A_{9}A_{9} \end{array}\right]$$

**Figure 7:**
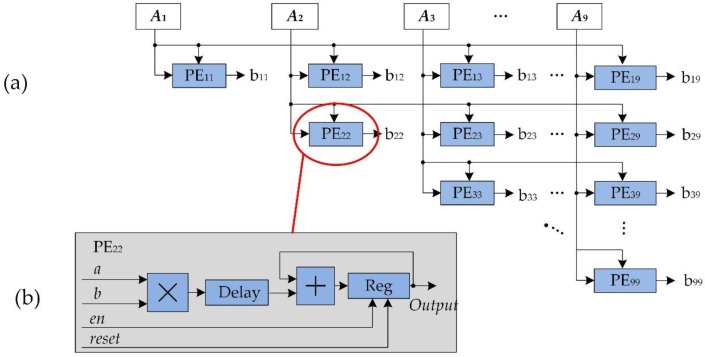
Parallel computation method for *A^T^A.* (**a**) is the matrix multiplication unit, (**b**) is multiplicative unit.

With considering the symmetry of *A^T^A*, an FPGA-based parallel computation for the upper triangular matrix of *A^T^A* is presented and depicted in Figure 7. As seem from Figure 7a, a number of processing elements (PEs) units are employed to reduce the complexity of computation [49]. All of the PE units are with the same structure, i.e., “*a*_1_*b*_1_ *+ a*_2_*b*_2_”, which is enlarged in Figure 7b. Similarly, the method for computation of *A^T^L* in Equation (8) is the same as that of the *A^T^A*.

#### 3.2.4. FPGA-Based Parallel Computation for *B*^−1^

Also due to limitation of the FPGA resources, computing matrix inversion, *B*^−1^ is implemented through Cholesky decomposition method [50], i.e.:

*B* = *LDL^T^* (11)

where *L* is a lower triangular matrix, *D* is a diagonal matrix, and *L^T^* is *L*’s transpose. The solutions of *L* and *D* are expressed by:

(12) $$\{\begin{array}{c} d_{11}=b_{11},d_{ii}=b_{ii}-\sum_{k=1}^{i-1}l_{ik}d_{kk}l_{ik}\;\;\;\;\;\;\;\;\;\;(2\leq i\leq n)\\ l_{i1}=\frac{b_{i1}}{d_{11}}(2\leq i\leq n),l_{ij}=\left(b_{ij}-\sum_{k=1}^{j-1}l_{ik}d_{kk}l_{jk}\right)/d_{jj}\;\;(1\leq j<i\leq n) \end{array}$$

With the characteristics of *LDL^T^*, *d*_11_ and *l_i_*_1_ are first calculated, and then *d_ii_* and *l_ij_* are calculated on the basis of *d*_11_ and *l_i_*_1_. In other words, the latter results are calculated on the basis of the formerly computed results. Since Equations (11) and (12) avoid the computation of square root and alleviate the dependency of the data, the modified equations, Equations (11) and (12), are able to speed up the computation [51].

**Figure 8:**
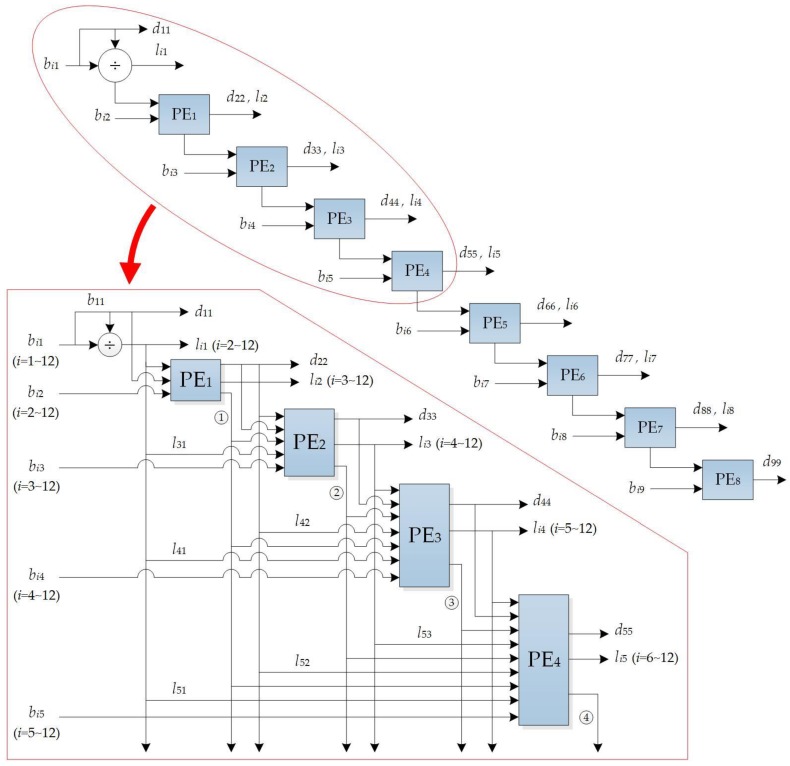
FPGA-based computation of *B*^−1^ through *LDL^T^* decomposition, where the dimension of *B* at 9 × 9 (i.e., *n* = 9) is taken as an example.

The FPGA-based parallel computation for the *LDL^T^* is depicted in Figure 8. As observed from Figure 8, it includes 1 driver and 8 PE units. The PE*_i_* (*i* = 1, 2, 3 and 4) are for calculating *d_ii_* and *l_ij_*. Thus, the computation of *B*^−1^ can be divided into two steps: (1) Decompose *B* into the *LDL^T^*; (2) Compute the inversion of *LDL^T^* by:

(13) $$B^{-1}={\left(LDL^{T}\right)}^{-1}={\left(L^{T}\right)}^{-1}{\left(LD\right)}^{-1}={\left(L^{-1}\right)}^{T}D^{-1}L^{-1}$$

**Figure 9:**
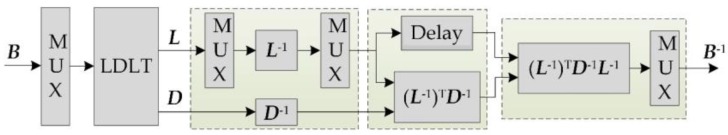
FPGA-based flowchart of *B*^−1^ computation.

Summarily, the FPGA-based flowchart of *B*^−1^ is depicted in Figure 9, which consists of five parts. Each of the parts is explained in detail as follows.

(1) The first MUX at the left hand of Figure 9 is to construct the column elements of *B*;
(2) The *LDL^T^* is to calculate the elements of *L* and *D*;
(3) The third dash-rectangle, consisting of two MUXes, *L*^−1^ and *D*^−1^, is to compute the inversion of *L* and *D*, where the second MUX is to construct the vector of *L*; *L*^−1^ presents the calculation of the inversion of *L* through unit lower triangular matrix [52]; the third MUX is to construct the vector of (*L*^−1^)*^T^*; and *D*^−1^ is to construct the row vectors composed of the elements of *D* and calculate the reciprocal of the elements of *D* by a divider; *D*^−1^ is the outputted result;
(4) (*L*^−1^)*^T^D*^−1^ module means that (*L*^−1^)*^T^* matrix multiplies with *D*^−1^;
(5) (*L*^−1^)*^T^D*^−1^*L*^−1^ module denotes that (*L*^−1^)*^T^D*^−1^ matrix multiplies with *L*^−1^;
(6) *B*^−1^ is the outputted result through the last MUX.

## 4. Experiments and Performance Analysis

### 4.1. Test Area and Data Set

**Figure 10:**
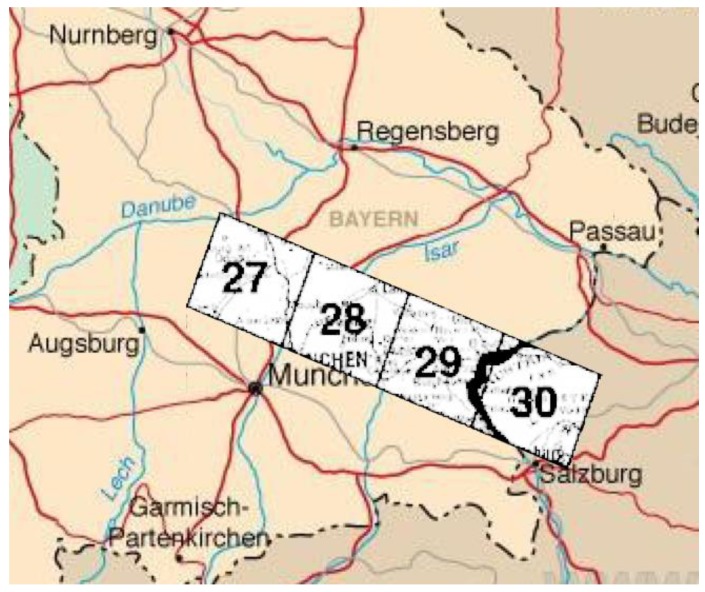
Geographical location of test area.

The test area and data set are from DLR, Germany. The data sets were used for the inflight geometric calibration of MOMS-2P. The test area is comprised of Scenes 27 through 30 from southeast of Germany to about 160 km beyond the Austrian border (see Figure 10, in which the area is about 178 × 50 km^2^, and the ground pixel size of imagery is 5.9 m at 390 km orbit height).

**Figure 11:**
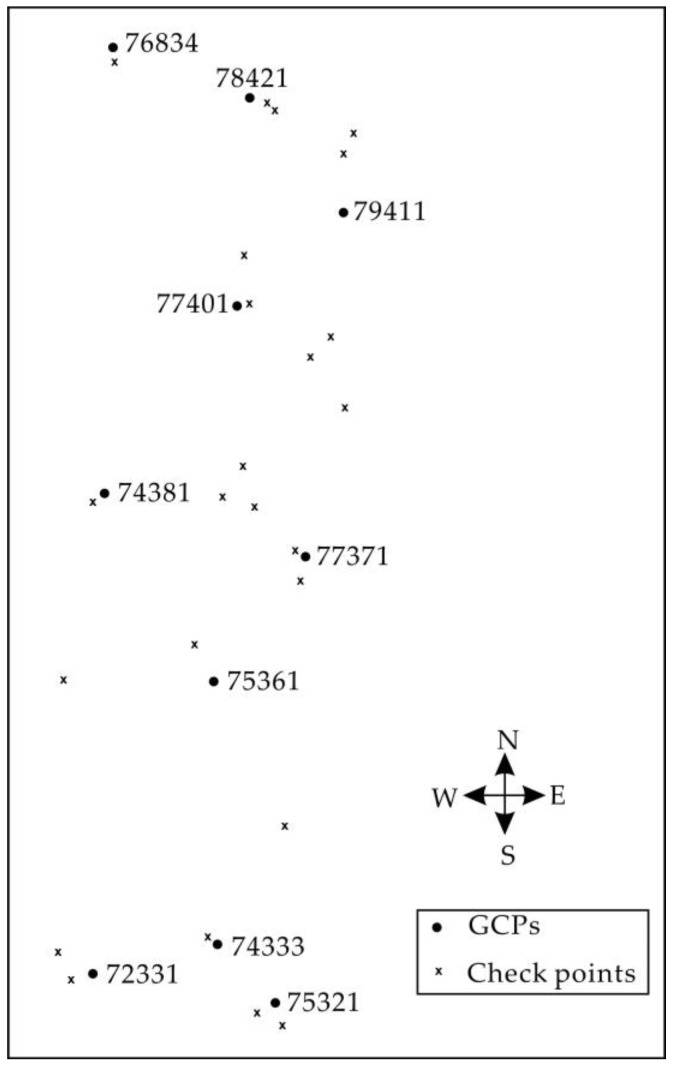
Distribution of 10 GCPs and check points in the test field.

In the test area, the ground coordinates of 10 GCPs and 24 check points were obtained by topographic maps at a scale of 1:50,000 with an accuracy of 1.5 m in *X*, *Y* and *Z* (see Figure 11, the details can be found in [46,47]).

They were located in the 4th zone of Gauss-Krueger coordinate system. The navigation data of Orientation Lines (OLs) (the definition of OLs can be found in [46,47]), the ground coordinates of GCPs and the corresponding image coordinates were all provided by Institute of Photogrammetry at University of Stuttgart [53].

### 4.2. Hardware Environments

A Virtex-7 FPGA VC707 board produced by Xilinx Corporation (San Jose, CA, USA). The key chip, XC7VX485T FFG1761, has 485,760 logic cells, 507,200 CLB Flip-Flops, 2800 DSP DSP48E1, 37,080 Kb total block RAM and 700 Single-Ended I/O. The Vivado software (v. 2014.2) and the System Generator software (v. 2014.2) developed by Xilinx Corporation are employed as the development tools. The hardware description language is Verilog HDL. A PC (personal computer) with a Windows 7 (64 bit) operation system is selected for the purpose of comparison analysis. The PC computer is equipped with an Intel(R) Core(TM) i7-4790 CPU @ 3.60 GHz and 8 GB RAM.

### 4.3. Analysis of Various Floating-Point Data Widths

#### 4.3.1. Relationship between the Data Width and the Accuracy

Due to the resource limitation of the FPGA chip, this paper attempts to study the relationship between the floating-point data width vs. the accuracy of geometric calibration. The experiment is conducted using 6 GCPs under the floating-point data widths, 44-bit, 48-bit, 50-bit, 54-bit and 64-bit. In accordance with the IEEE standard 754, a floating-point data width consists of the sign part, exponent part and fractional part. The five types of floating-point data widths are experimented, as shown in Table 2.

With the different data widths, the solutions of the coefficients in Equation (5) computed by the FPGA and the PC computer are presented in Table 3. As seen in Table 3, when the data width increases from 44-bit to 64-bit, the maximum differences of the coefficients, Δ*b*_0_ and Δ*c*_0_ decrease from 2.8684 to 5.8651 × 10^−4^, and from 0.9676 to 0.0021, respectively. The differences of the coefficients, Δ*d*_0_, Δ*e*_0_, Δ*f*_0_, Δ*d*_1_, Δ*e*_1_ and Δ*f*_1_, decrease from approximatively 1 × 10^−7^ to 1 × 10^−10^ when the data wide decrease from 44-bit to 64-bit. When the data width reaches 64-bit, the solutions of 9 coefficients solved by the FPGA and by the PC computer are almost exactly the same. Consequently, the experimental results discover that (1) the differences of geometric calibration parameters solved by FPGA and PC computer are small enough; (2) With increasing the data widths, the accuracy of geometric calibration parameters increases.

#### 4.3.2. Relationship between the Data Width and the Consumption of FPGA Resources

**Figure 12:**
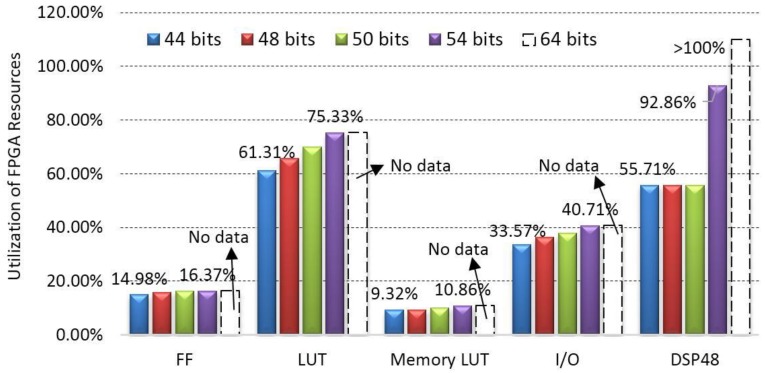
Comparison analysis of the utilizations of several types of FPGA resources vs. different widths of data.

Although an increasing data width can increase the accuracy of the calibration parameters, it consumes more FPGA resources. Thereby, it is necessary to investigate the relationship between the data width and the consumption of FPGA resources. For this reason, the five widths of floating-point data and the consumptions of FPGA resources, including FF, LUT, Memory LUT, I/O and DSP48, are analyzed. The results are displayed in Figure 12. As seen from Figure 12, when the data width increases from 44-bit to 54-bit, the utilization of FF increases from 14.98% to 16.37%, LUT from 61.31% to 75.33%, Memory LUT from 9.32% to 10.86%, I/O from 33.57% to 40.71% and DSP48 from 55.71% to 92.86%. The consumption of DSP48 unit increases from 6 to 10 when data width changes from 50-bit to 54-bit. This means that the DSP48 resources consume very fast when data width increases. When the data width reaches 64-bit, the utilizations of FPGA resources reaches 100%. It can therefore be concluded that the data width with 54-bit is recommended in this paper.

#### 4.3.3. Relationship between the Data Width and the Computational Speed

Computational speed is considered as one of the most important indexes in FPGA-based implementation. This sub-section discusses the relationship between the data width and the computational speed using the five types of floating-point data. For the purpose of comparison analysis, the calibration parameters are also computed by PC-based Microsoft Visual studio 2015 (C++). The results are presented in Table 3 and Table 4. As observed from Table 3 and Table 4, the computational speed at data widths of 44-bit, 48-bit, 50-bit and 54-bit can reach a 217 clock cycle (approximately 0.01736 ms under the clock frequency of 12.5 MHz) when using FPGA, which is 22 times faster than that by the PC-based implementation. However, the computation at the data width of 64-bit is failure to be operated, since the utilization of the DSP48 unit reaches 100% (see Figure 12). Hence, the data width with 64-bit is not recommended.

With the experimental results in Table 3, it can be concluded that when the data width increases from 44-bit to 64-bit, the accuracy can be improved, while the speed of FPGA-based implementation remains consistent, except for the data width of 64-bit. The reasons are that (1) the IP cores with different data widths can be defined as the same clock delay for the results outputting; (2) the larger data width will consume more DSP48 unit, resulting in that the consumption of DSP48 in 64-bit data width reaches over 100% (see Figure 12). Therefore, a width of floating point data with 50-bit is recommended for on-board geometric calibration.

### 4.4. Analysis of the Optimum Number of GCPs

#### 4.4.1. Relationship between the Number of GCPs and the Accuracy

In general, the more GCPs, the higher geometric calibration accuracy. However, for a FPGA-based implementation, many GCPs will add the burden of computational time and the consumption of FPGA resources. Thereby, it is necessary to investigate the optimum number of GCPs for on-board geometric calibration. For this reason, this sub-section investigates the different numbers of GCPs (i.e., 5, 6, 8 and 10) vs. computational accuracy when the floating-point data width is fixed at 50-bit. Also for the purpose of comparison, the calibration parameters solved by PC-based Microsoft Visual studio 2015 (C++) is applied as the references under the same GCPs. The differences of the parameters between the FPGA-based and the PC-based computations are shown in Table 5. As seen in Table 5, with increasing number of GCPs, the differences of calibration parameters become smaller and smaller. The maximum difference reaches 0.015 for *a*_0_, when the number of GCPs is 5; even decreases to 0.008 when the number of GCPs reaches 10. The differences of the other parameters, *b*_0_, *c*_0_, *d*_0_, *e*_0_, *f*_0_, *d*_1_, *e*_1_ and *f*_1_ in Table 5, display a similar phenomenon.

#### 4.4.2. Relationship between the Number of GCPs and the Consumption of FPGA Resources

In order to select an optimum number of GCPs, a relationship between the number of GCPs and the consumption of the FPGA resources is investigated. With the fixed data wide at 50 bit as described above, 5 GCPs, 6 GCPs, 8 GCPs and 10 GCPs are selected to investigate the consumption of FPGA resources, respectively. The results are presented in Table 6. As observed from Table 6, when the number of GCPs varies from 5 to 10, the consumptions of the BRAM, DSP48 and BUFG remain the same, but the consumptions of FF, LUT and Memory LUT increase a little bit. This means that the increasing number of GCPs will not significantly consume the FPGA resources.

#### 4.4.3. Relationship between the Number of GCPs and the Computational Speed

From Vivado software’s instruction, the clock cycles span from 75.94 ns, 76.76 ns, 75.34 ns to 75.82 ns. To ensure enough clock delay when executing a floating-point calculation, the clock cycle is set at 80 ns (the corresponding clock frequency is 12.5 MHz) in this group of experiments. The computational time under the different number of GCPs is listed in Table 7. As observed in Table 7, the computational time changes from 0.0170 ms to 0.0186 ms when the number of GCPs increases from 5 to 10, while the computational time from PC-based computation increases from 0.377 ms to 0.463 ms. In other words, the FPGA-based maximum computation speed can reach approximately 22 times faster than the PC-based does when five GCPs are adopted; the FPGA-based minimum computation speedup can achieve approximately 25 times faster than the PC-based does when the number of GCPs increases to 10. Thereby, it can be concluded that FPGA-based on-board implementation of geometric calibration can reach approximately 24 times faster than the PC-based does.

### 4.5. Accuracy Comparison between FPGA-Based and Inflight-Based Computations

To investigate the relationship between FPGA-based and inflight-based implementations of geometric calibration, the data wide is fixed at 50 bits; the 10 GCPs and 24 check points are selected; and the calibration model, associated with other conditions, are the same. The experimental results are presented in Table 8. As shown in Table 8, the *RMS_X_* of 11 m for *X*-coordinate, *RMS_Y_* of 8 m for *Y*-coordinate and *RMS_Z_* of 11 m for *Z*-coordinate can be reached. The *RMS* differences (*noted*, ∆) between the inflight-based and the FPGA-based implementations are 0.16 m for *X*-coordinate, 0.19 m for the *Y*-coordinate and 0.11 m for the *Z*-coordinate, respectively.

## 5. Conclusions

This paper first presents an FPGA-based on-board computation and implementation for geometric calibration. A Xilinx Virtex-7 FPGA VC707 board is selected as hardware and the experimental data used for inflight geometric calibration from DLR (Köln, Germany), is employed to validate our method. The main contributions of this paper are as follows.

(1) An FPGA-based on-board geometric calibration computation is designed and implemented.
(2) FPGA-based parallel computation for coefficient matrixes construction (e.g., matrix *A* and matrix *L*), matrix multiplication (e.g., *A^T^A* and *A^T^L*), matrix decomposition (e.g., *B* = *LDL^T^*), and matrix inversion (e.g., *B*^−1^) are developed. With the experimental results, it has been demonstrated that the proposed method is able to save large amount of the FPGA resources.
(3) From the experimental results, it can be found that:
  (a) With increasing data width, FPGA resources consume increasingly. For example, the utilization of DSP48 unit suddenly increase from 55.7% to 92.9%, this fact demonstrates that the data width of 64-bit is impropriate for on-board implementation of geometric calibration for the selected FPGA chip due to the limitation of the FPGA resource.
  (b) The computation speed executed by the FPGA is approximately 22 times faster than that executed by the PC computer when five GCPs are adopted; and approximately 25 times faster than that executed by the PC computer when 10 GCPs are adopted. It can therefore be concluded that the computing speed executed by the FPGA can reach approximately 24 times faster than that executed by PC computer with Microsoft Visual Studio 2015 (C++).
  (c) More than 90% of the DSP48 unit resources are consumed when the data width of 54-bit or 64-bit is selected. Considering the limitation of FPGA resources, a width of 50-bit floating point data is recommended, as which it meets the requirement of geometric calibration accuracy.
  (d) When the floating-point data width is fixed at 50-bit and the number of GCPs varies from five to 10, the utilizations of the FPGA’s BRAM, DSP48, and BUFG remain unchanged, the utilizations of FF, LUT and Memory LUT slightly increase and the computational speed increase a little bit. This means that increasing the number of GCPs will not significantly increase the consumption of the FPGA resources and the computational speed. Therefore, six GCPs are recommended during an on-board geometric calibration.

The proposed method in this paper not only can be used for satellites, but also used for van-based mobile mapping and ground-based robots for speedup of geometric calibration. Moreover, part of the FPGA-based modules developed in this paper, such as matrix multiplication and matrix inversion, can be used for geometric calibration captured by frame cameras as well.

## Figures and Tables

**Table 1:** Lab-calibrated camera parameters for moms-2p [46,48].

	HR5A	HR5B	ST6	ST7
*f* (mm)	660.256	660.224	237.241	237.246
*x*_0_ (pixel)	0.1	0.2	−7.2	−0.5
*y*_0_ (pixel)	−0.4	0.1	8.0	19.2
*Kc* (pixel)	−0.3	−0.4	−1.1	1.7
*k* (mdeg)	−2.9	5.4	−1.5	−1.4

**Table 2:** Format of floating-point.

	44-Bit	48-Bit	50-Bit	54-Bit	64-Bit
Sign part	1	1	1	1	1
Exponent part	8	8	9	9	11
Fractional part	35	39	40	44	52

**Table 3:** Differences of the results computed by FPGA-based and PC-based computations with various data width.

|PC-FPGA|	44-Bit	48-Bit	50-Bit	54-Bit	64-Bit
Δ*a*_0_	0.1026	0.0408	0.0050	4.3282 × 10^−4^	1.8679 × 10^−5^
Δ*b*_0_	2.8684	0.1184	0.0418	0.0019	5.8651 × 10^−4^
Δ*c*_0_	0.9676	0.0301	0.0043	0.0018	0.0021
Δ*d*_0_	2.018 × 10^−7^	1.995 × 10^−8^	2.281 × 10^−9^	9.935 × 10^−10^	8.365 × 10^−10^
Δ*e*_0_	3.359 × 10^−7^	1.031 × 10^−8^	1.154 × 10^−9^	5.487 × 10^−11^	7.704 × 10^−11^
Δ*f*_0_	7.660 × 10^−7^	2.798 × 10^−8^	3.685 × 10^−9^	4.254 × 10^−10^	1.630 × 10^−10^
Δ*d*_1_	1.316 × 10^−8^	6.464 × 10^−10^	1.685 × 10^−10^	1.819 × 10^−12^	5.190 × 10^−12^
Δ*e*_1_	9.478 × 10^−11^	7.295 × 10^−11^	7.087 × 10^−12^	1.687 × 10^−12^	3.684 × 10^−13^
Δ*f*_1_	2.776 × 10^−8^	1.912 × 10^−9^	1.833 × 10^−10^	9.606 × 10^−12^	8.882 × 10^−14^

**Table 4:** Comparision of FPGA-based and PC-based computing time.

FPGA-Based Implementation		PC-Based Implementation (64-Bit)	Conclusion
44-bit	0.0174 (ms) (217 clock, 12.5 MHz)	0.378 (ms)	The FPGA-based computation is 22 times faster than the PC-based does.
48-bit			
50-bit			
54-bit			
64-bit	Fail to calculation		Fail to calculation

**Table 5:** Differences of calibration between FPGA-based and PC-based calculations.

|PC-FPGA|	5 GCPs	6 GCPs	8 GCPs	10 GCPs
Δ*a*_0_	0.01481804	0.00502983	0.00648983	0.00749144
Δ*b*_0_	1.12103 × 10^−5^	0.04178977	0.00766325	0.00091729
Δ*c*_0_	0.00077522	0.00427705	0.00244571	0.00394770
Δ*d*_0_	3.3568 × 10^−9^	2.28135 × 10^−9^	2.19579 × 10^−10^	2.72209 × 10^−10^
Δ*e*_0_	2.99778 × 10^−10^	1.15354 × 10^−9^	1.19552 × 10^−9^	5.38698 × 10^−10^
Δ*f*_0_	1.5894 × 10^−9^	3.68499 × 10^−9^	7.32229 × 10^−10^	2.71596 × 10^−9^
Δ*d*_1_	5.21121 × 10^−11^	1.68484 × 10^−10^	1.68234 × 10^−11^	3.37348 × 10^−11^
Δ*e*_1_	1.39511 × 10^−11^	7.08677 × 10^−12^	1.05921 × 10^−11^	1.29294 × 10^−11^
Δ*f*_1_	9.92273 × 10^−12^	1.8332 × 10^−10^	9.54881 × 10^−11^	1.29121 × 10^−10^

**Table 6:** The utilizations of FPGA resources under different numbers of GCPS.

GCPs	BRAM (%)	DSP48 (%)	BUFG (%)	FF (%)	LUT (%)	Memory LUT (%)
5	1.31	55.71	3.12	16.14	69.67	9.46
6	1.31	55.71	3.12	16.37	70.04	10.09
8	1.31	55.71	3.12	16.96	70.63	11.48
10	1.31	55.71	3.12	17.58	71.78	12.92

**Table 7:** The FPGA-based computational time under different numbers of GCPS.

GCPs	By FPGA (ms)	By PC (ms)	Speedup
5	0.0170 (213 clock, 12.5 MHz)	0.377	22
6	0.0174 (217 clock, 12.5 MHz)	0.378	22
8	0.0180 (225 clock, 12.5 MHz)	0.458	25
10	0.0186 (233 clock, 12.5 MHz)	0.463	25

**Table 8:** Accuracy of ground coordinates calculated by inflight-based and FPGA-based implements.

	GCP	Check Points	*σ*_0_ [um]	*σ_x_* [m]	*σ_y_* [m]	*σ_z_* [m]	*RMS_X_* [m]	*RMS_Y_* [m]	*RMS_Z_* [m]
Inflight-based [43]	10	24	6.99	7.14	5.32	6.01	11.14	8.28	10.76
FPGA-based	10	24	6.99	7.29	5.49	6.16	11.31	8.47	10.87
∆				0.15	0.17	0.15	0.16	0.19	0.11
